# Biographical Feature: In memoriam Jay A. Nelson (1948–2024)

**DOI:** 10.1128/jvi.01930-24

**Published:** 2024-12-10

**Authors:** Meaghan H. Hancock

**Affiliations:** 1 Oregon Health and Science University 56870 , Beaverton, Oregon, USA; The University of Arizona, Tucson, Arizona, USA

**Keywords:** cytomegalovirus, flavivirus, human immunodeficiency virus

## TEXT

**Figure:**
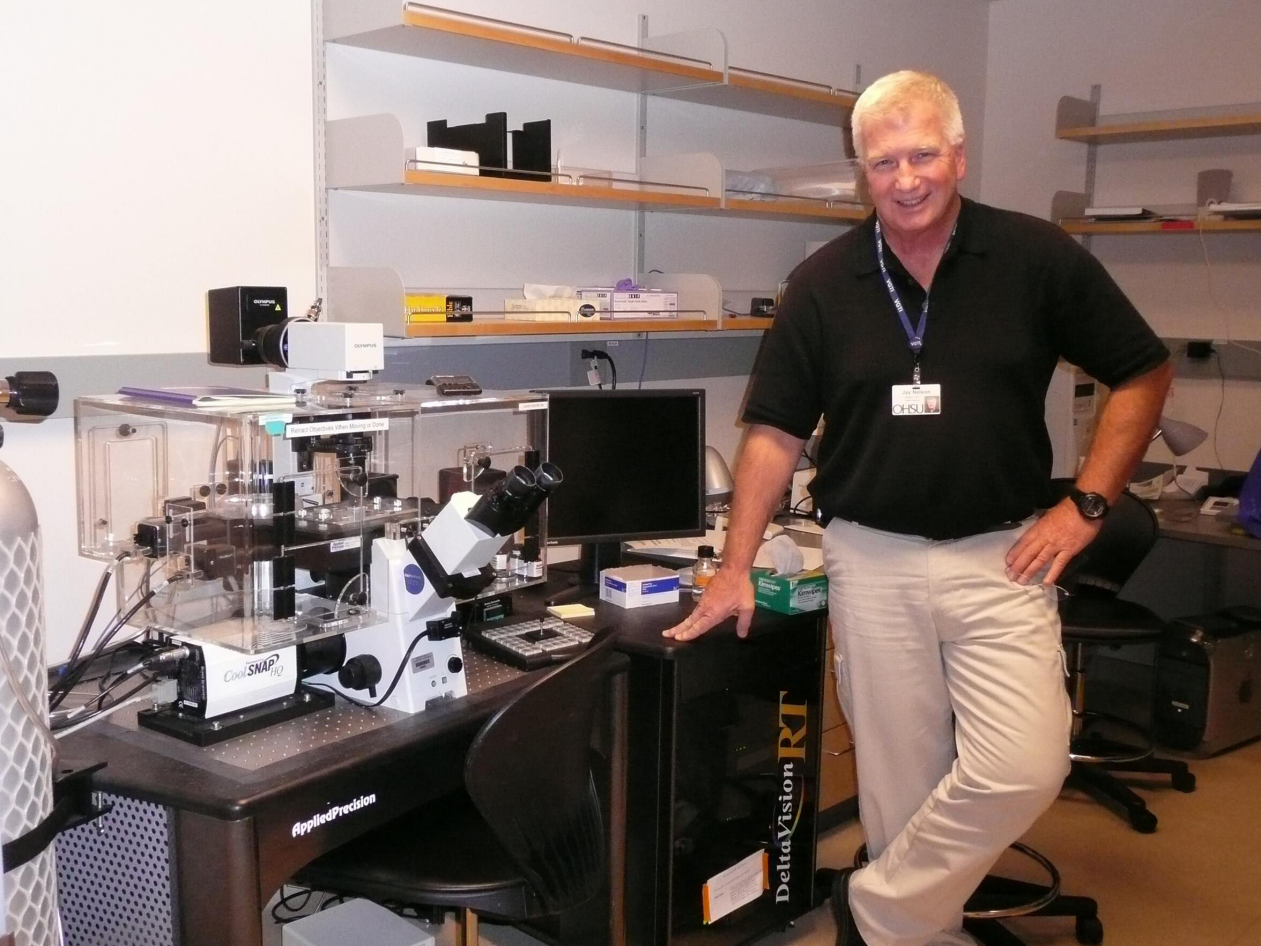


Jay A. Nelson, a dedicated scientist, mentor, and friend, died 20 January 2024. Jay’s impact on the virology community was profound; he was a constant source of support, offered thoughtful critiques, insightful brainstorming sessions, and generous letters of support, and was someone you could always turn to for help or advice. He cared deeply about the careers and the lives of those he worked with, and this will be forever remembered by his colleagues and friends.

Jay received his bachelor’s degree in Biological Sciences at California State University in 1975. From there he moved to Oregon State University (OSU) and was awarded his Ph.D. in Microbiology working with Drs. Jo-Ann Leong and Jay Levy studying particles with characteristics of retroviruses in human placenta. This time in Jay’s career required balancing a young family with the rigorous demands of graduate student life, including obtaining and processing placental tissues at all hours of the night and day. Jay always fondly remembered his time at OSU and cultivated a deep and lasting friendship with his mentor Jo-Ann Leong and her family that continued until his death. Jay stayed in the Pacific Northwest to complete his Postdoctoral training at the Fred Hutchinson Cancer Research Center and the University of Washington where he began his long and productive career studying cytomegaloviruses (CMVs) with Drs. Denise Galloway and John McDougall. Following this time, Jay moved to Scripps Research Institute and worked alongside Dr. Michael Oldstone as an Assistant and Associate Professor and studied, among many topics, co-infections with human immunodeficiency virus (HIV) and CMV. It was during his time at Scripps that Jay and his colleagues performed seminal research into the CMV major immediate early promoter (MIEP). Together with Dr. Peter Ghazal, they found that the human cytomegalovirus (HCMV) MIEP is not a pan-specific promoter and that the majority of tissues expressing the MIEP in mice correlate with tissues naturally infected by the virus in the human host.

In 1992, Jay was recruited to Oregon Health and Science University (OHSU) as a Professor where he spent the remainder of his career. Working at OHSU allowed Jay the freedom to pursue his interests in the molecular pathogenesis of CMV infection. He and colleagues initially characterized interactions between CMV and myeloid and endothelial cells as well as developed the first humanized mouse model for studying HCMV latency and reactivation *in vivo*. In 2000, Jay became the founder and Director of the Vaccine and Gene Therapy Institute (VGTI) at OHSU and later, VGTI-Florida. Jay, along with Assistant Director Dr. Louis Picker, recruited scientists from the fields of virology, immunology, vaccinology, and primate biology to center the Institute on solving complex biological problems using non-human primate models. The VGTI remains a highly collaborative, innovative, and successful center for scientific research to this day thanks to the forethought and vision of Jay Nelson. To honor Jay’s commitment and dedication to the mission of VGTI, the building housing VGTI has been named the Jay Nelson Research Building.

One of Jay’s biggest strengths was his ability to bring together scientists to address big-picture problems. Jay set high bars in the field for rigor and excellence, and this served as the basis for all his leadership. Jay was the Director of the Pacific Northwest Regional Centers for Excellence in Biodefense and Emerging Diseases, a consortium of scientists with extensive expertise in basic and translational research directed at a broad range of NIAID Category A-C Priority Pathogens with an emphasis on flaviviruses, including dengue, West Nile, and Japanese encephalitis viruses. Additionally, Jay and Dr. Michael Gale were Principal Investigators (PIs) on an NIAID contract to develop small molecule agonists of cellular IRFs as vaccine adjuvants. Jay was also the PI of a program project grant studying the molecular mechanisms used by HCMV during latency establishment and reactivation. This productive research consortium fed Jay’s ongoing passion for CMV biology. Monthly meetings often contained lively, deep, and sometimes philosophical discussions of how to define and measure CMV persistence and latency, and all in attendance learned from Jay’s vast knowledge of the field. Jay also had a unique ability to steer collaborators towards a common goal which resulted in lasting impactful insights into CMV proteins and non-coding RNAs and their effect on myeloid cell infection. Altogether, Jay published more than 200 papers in peer-reviewed and high-impact journals, including but not limited to *Science*, *Nature*, *Cell*, *Lancet*, *PNAS*, *Cell Host & Microbe*, *PLoS Pathogens*, and *Journal of Virology*.

Jay’s exceptional talent for fostering interdisciplinary collaboration is perhaps best exemplified by the successful development and characterization of CMV-based vaccine vectors for HIV, TB, and a number of other infectious diseases. Jay’s deep understanding of CMV biology has significantly aided in understanding the unique and efficacious T cell responses elicited by genetically modified strains of CMV whose impact will be further realized by the continued research of his colleagues at the VGTI and around the globe.

Significantly, Jay always demonstrated a deep commitment to mentorship, exemplified by his training more than 40 graduate students, postdoctoral, and research fellows over his career, many of whom he remained in close contact with until his death. Furthermore, he, along with Dr. David Johnson, played an instrumental role in the success of the International Herpesvirus Workshop (IHW), a yearly gathering of herpesvirologists from around the world. Each year the meeting is held in a different location either nationally or internationally, and Jay dedicated himself to writing the NIH grants needed to support trainee travel to the workshop. In tribute to Jay’s dedication to IHW trainees, friends and colleagues have established an endowment to sponsor graduate student and postdoctoral trainee attendance at future meetings (donate.ohsufoundation.org/jay-nelson).

Beyond his mentorship, Jay dedicated significant time to advancing the field of virology through teaching, administrative roles, consultations, invited lectures, and service on grant review panels for the NIH, American Cancer Society, and the American Foundation for AIDS Research. In addition, Jay was an Associate Editor with *PLoS Pathogens* and an Editor of *Journal of Virology*. He earned numerous awards and distinctions during his career, including Faculty awards from the American Cancer Society and the Weller Lifetime Award for Excellence in Cytomegalovirus Research, and was a Fellow of the American Academy of Microbiology.

Jay was a kind and loving man, deeply committed to his family and friends, yet possessed a fiercely competitive spirit. Outside of his professional life, Jay found joy in simple pleasures: playing ball with his dogs, card games (especially cribbage), fly fishing, and travel, all pursuits that reflected his creativity and adventurous spirit. His enthusiastic approach to life and zest for living inspired all who knew him to cherish each day. Like all great scientists, Jay’s legacy will live on not only through his contributions to the field of virology but also the impact he had personally on those of us closest to him.

